# Baicalin–Myricetin-Coated Selenium Nanoparticles Mitigate Pathology in an Aβ1-42 Mice Model of Alzheimer’s Disease

**DOI:** 10.3390/ph18091391

**Published:** 2025-09-17

**Authors:** Rosa Martha Pérez Gutiérrez, Julio Téllez Gómez, José María Mota Flores, Mónica Corea Téllez, Alethia Muñiz Ramírez

**Affiliations:** 1 Laboratorio de Investigación de Productos Naturales, Escuela Superior de Ingeniería Química e Industrias Extractivas, Instituto Politécnico Nacional (IPN) Unidad Profesional Adolfo López ateos S/N Av., Instituto Politécnico Nacional, Ciudad de México 07708, Mexico; julio_tellez_gomez@hotmail.com (J.T.G.); josemariamota@yahoo.com (J.M.M.F.); 2 Laboratorio de Investigación en Polímeros y Nanomateriales Escuela Superior de Ingeniería Química e Industrias Extractivas, Instituto Politécnico Nacional (IPN) Unidad Profesional Adolfo López Mateos S/N Av., Instituto Politécnico Nacional, Ciudad de México 07708, Mexico; mcoreat@yahoo.com.mx; 3 SECIHTI-Instituto Potosino de Investigación Científica y Tecnológica (IPICYT), División de Materiales Avanzados, Camino a la Presa San José 2055, Lomas 4th Section, San Luis Potosí 78216, Mexico

**Keywords:** nanoparticles, baicalin–myricetin, amyloid Aβ1-42, behavior test, neuroinflammation

## Abstract

**Background**: Current Alzheimer’s disease (AD) treatments primarily focus on symptom management and offer limited potential to arrest disease progression. To address this limitation, we developed baicalin–myricetin (BM) functionalized selenium nanoparticles (SeNPs), termed BMSe@BSA, aimed at multi-targeted neuroprotection. **Materials and Methods**: BMSe@BSA nanoparticles were synthesized via a gel–sol technique using bovine serum albumin (BSA), ascorbic acid, selenous acid, and BM. Interactions among BSA, BM, and SeNPs were characterized by microscopy and spectrometry. Cytotoxicity was assessed on RAW 264.7 and PC12 cells to determine biocompatibility. Neuroinflammation and cognitive function were evaluated in C57BL6/J mice challenged with Aβ1-42. Recognition memory was tested through open-field exploration, novel object recognition (NOR), and T-maze assays. Inflammatory markers (IL-1β and TNF-α) and microglial changes in the cerebral cortex were quantified, while amyloid fibril morphology was assessed using atomic force microscopy (AFM). **Results**: Spectroscopic analysis verified successful BM functionalization. Transmission electron microscopy revealed a spherical morphology with an average particle size of 90.57 nm, zeta potential of 27.2 mV, and a polydispersity index (PDI) of 0.270. BM entrapment efficiency reached approximately 90%. Cytotoxicity assays confirmed the nanoparticles’ safety, with no toxicity observed at concentrations up to 400 µg/mL after 4 h of incubation. BMSe@BSA effectively inhibited amyloid fibril formation, downregulated pro-inflammatory cytokine expression, preserved neuronal integrity, and significantly enhanced cognitive performance in AD mouse models. Conclusion: BMSe@BSA appear as a potential nanotherapeutic approach for targeted brain delivery and multi-pathway intervention in Alzheimer’s disease.

## 1. Introduction

Alzheimer’s disease (AD) is the most prevalent form of dementia and represents one of the most pressing global health challenges. In the United States, it has become the third leading cause of death. Although its etiology remains partially understood, genetic factors contribute to approximately 5–10% of cases classified as familial AD, while the remaining 90–95% are considered sporadic. Possession of the ApoE ε4 allele—either in heterozygous or homozygous form—markedly elevates the risk of developing the disease [1]. Alzheimer’s disease (AD) affects over 40 million individuals globally, with its prevalence doubling approximately every five years after the age of 65 [2]. While age-related memory decline is considered a normal process that does not interfere with daily functioning, AD is characterized by a progressive and accelerated cognitive deterioration that significantly impairs quality of life. Mild cognitive impairment (MCI), a condition often preceding AD, is reported to affect more than 40% of individuals over the age of 60 [3]. AD imposes a substantial burden on patients, reflected in the loss of healthy life years due to disability and increased risk of premature death The disease also places considerable strain on caregivers, with caregiver burden intensifying in parallel with disease severity.

Epidemiological data on diabetes revealed that global prevalence: In 2021, 536.6 million adults (10.5%) aged 20–79 had diabetes. This is projected to rise to 783.2 million (12.2%) by 2045. Growth Trends: Between 1990 and 2021, global prevalence nearly doubled—from 3.09% to 6.11% [4]. Socioeconomic burden of diabetes indicated that diabetes cost the world USD 966 billion in 2021, projected to exceed USD 1 trillion by 2030 and National Health Budgets: In Latin America, diabetes consumes 7–15% of total health expenditures [5].

Early-stage AD brings more noticeable memory deficits, such as forgetting familiar names and events, and confusion in unfamiliar situations. Ultimately, late-stage AD patients may be non-verbal or incoherent, have severe sleep and motor deficits, and become increasingly aggressive, paranoid, or unresponsive. An emerging, frightening theme from recent studies is that by the time cognitive impairment is noticed by a patient, their close relations, or doctor, the cascade of events leading to full-blown AD may be irreversible without a disease-modifying therapy [6]. AD progression can be divided into three successive stages: (1) preclinical AD (no cognitive impairment based on standard assessment or AD biomarkers), (2) mild cognitive impairment (MCI) due to AD (impairment of memory or other cognitive domains based on standard assessment and evidence of AD biomarkers), and (3) dementia due to AD (dementia and evidence of AD biomarkers) Progressive neuronal loss and irreversible cognitive decline typically occur during these stages [7].

To date, non-demented individuals exhibiting β-amyloid (Aβ) plaque pathology are considered to display neuropathological features consistent with Alzheimer’s disease (AD) [8]. These individuals are classified as pathologically diagnosed preclinical AD (p-pre-AD) cases [9]. In contrast, individuals lacking Aβ plaques are categorized as non-AD cases, regardless of the presence or absence of neurofibrillary tangles (NFTs). NFTs, however, may occur independently of Aβ pathology in the brains of cognitively normal or impaired elderly individuals—a phenomenon known as primary age-related tauopathy (PART) [10]. By definition, PART does not involve the frontal or occipital cortices, making these regions particularly suitable for investigating AD-related proteomic alterations without confounding effects from PART.

The formation of Aβ plaques and NFTs in AD is associated with widespread changes in the brain proteome [11]. However, prior studies have either defined asymptomatic AD as non-demented individuals with moderate to severe NFT and neurotic plaque pathology or excluded such cases altogether. Consequently, individuals with early-stage Aβ and NFT pathology have often been misclassified as non-AD controls. This has left unresolved whether initial proteomic changes are detectable in p-pre-AD cases and how these changes compare to those observed in symptomatic stages of AD.

Although no cure for dementia currently exists, several cognition-enhancing drugs have been approved by the U.S. Food and Drug Administration (FDA) to alleviate the symptoms of Alzheimer’s disease. These medications aim primarily to stabilize cognitive and functional abilities, with a secondary benefit of potentially mitigating behavioral disturbances associated with dementia. Two main drug classes are approved for the treatment of Alzheimer’s disease: cholinesterase inhibitors and N-methyl-D-aspartate (NMDA) receptor antagonists [12]. Cholinesterase inhibitors function by reversibly binding to and inactivating acetylcholinesterase, thereby prolonging the presence of acetylcholine in the synaptic cleft. The three FDA-approved cholinesterase inhibitors are donepezil, galantamine, and rivastigmine [13]. Tacrine, the first drug in this class to receive approval, was subsequently withdrawn from the U.S. market due to reports of severe hepatotoxicity. Among these, only donepezil is approved for use in severe Alzheimer’s disease. Rivastigmine also carries an indication for mild to moderate dementia associated with Parkinson’s disease. Off-label use of cholinesterase inhibitors is common for other dementia types, including vascular dementia, mixed dementia, and dementia with Lewy bodies [14]. Memantine, the sole FDA-approved NMDA receptor antagonist, reduces neuronal calcium influx, thereby limiting excitotoxicity. It is indicated for moderate to severe Alzheimer’s disease. In December 2014, the FDA approved a combination capsule containing donepezil and memantine for the treatment of Alzheimer’s symptoms [15]. More recently, two anti-amyloid beta (anti-Aβ) therapies—ecanemab (approved in 2021) and lecanemab (approved in 2023)—have demonstrated biomarker modulation and delayed disease progression in Alzheimer’s patients, albeit with an increased incidence of adverse effects [16]. Additionally, novel disease-modifying therapies (DMTs), which aim to alter the course of Alzheimer’s rather than merely address symptoms, have entered Phase II and III clinical development between 2021 and 2024. A comprehensive review of the U.S. government clinical trial database identified 14 agents in Phase I, 34 in Phase II, and 11 in Phase III trials, with potential completion timelines extending to 2028 [17].

Natural compounds are gaining increasing attention as viable alternatives to synthetic therapies, which often entail considerable side effects. Among these, flavonoids stand out due to their ability to cross the blood–brain barrier, positioning them as promising agents for neurological intervention. A growing body of research has documented the neuroprotective effects of flavonoids in various neurodegenerative conditions, including Alzheimer’s disease (AD), Parkinson’s disease, and Huntington’s disease [18]. Despite ongoing research efforts, there is still no pharmaceutical treatment capable of definitively halting or reversing the progression of AD [19]. This challenge has spurred interest in naturally derived compounds with therapeutic potential. Many of these substances are not only easily extractable and mechanistically well understood but also boast favorable safety profiles [20]. Their abundance in fruits and vegetables further highlights their accessibility and ease of incorporation into the human diet, strengthening their relevance in maintaining brain health. Baicalin has demonstrated efficacy in mitigating memory deficits, as evidenced by performance improvements in the Morris water maze test. Its anti-neuroinflammatory properties are reflected in reduced glial cell activation and suppressed levels of proinflammatory cytokines, including IL-6 and TNF-α [21]. Furthermore, Baicalin promotes neurogenesis by increasing the population of astrocytes and neurons, along with enhancing the expression of neuronal markers such as neuron-specific enolase (NSE) and Nestin [22]. Similarly, Myricetin (M) has shown significant improvement in memory and learning abilities in animal models of Alzheimer’s disease [22]. By forming stable semiquinone radicals upon interaction with reactive species, it disrupts radical chain reactions and contributes to neuroprotection. In addition, Myricetin enhances cognitive performance in mice through the inhibition of acetylcholinesterase (AChE) activity [23].

In nature, selenium compounds occur in four primary oxidation states: elemental selenium (Se^0^), selenide (Se^2−^), selenite (Se^4+^), and selenate (Se^6+^). Among these, only elemental selenium (Se^0^) is non-toxic and water-soluble [24]. Due to the diverse oxidation states of selenium, its toxicity can be mitigated through nanosizing. The toxicity of each selenium species is influenced by its aqueous solubility and bioavailability. Selenium also exists in various chemical forms, including organic compounds such as selenium-methyl-selenocysteine, selenocysteine, and selenomethionine, as well as inorganic species like selenate and selenite. Selenium nanoparticles (SeNPs) exhibit notably lower toxicity compared to other forms and significantly enhance selenoprotein activity [25]. Owing to their selective targeting of both healthy and diseased cells, SeNPs can serve as therapeutic agents for a range of conditions including liver injury, antimicrobial resistance, diabetes, and Alzheimer’s disease. This targeted delivery minimizes side effects and ensures precise transport to specific tissues [26].

Another critical property of selenium nanoparticles (SeNPs) is their capacity to counteract drug-induced toxicity. Studies using zebrafish—organisms sharing approximately 85% of the human genome—demonstrate that SeNPs at low concentrations (5–10 µg/mL) do not cause significant mortality [27]. However, exposure to higher levels (20–25 µg/mL) results in marked physiological abnormalities and increased mortality rates. Similarly, experiments with rat dermal fibroblasts revealed an IC50 value of 46.5 µg/mL, indicating the concentration at which SeNPs inhibit a specific biological function by 50%. Interestingly, doses below 31 µg/mL were found to enhance cell viability, suggesting that SeNPs can be either toxic or beneficial depending on the administered concentration [28]. At lower doses, SeNPs are efficiently metabolized by the liver and kidneys. In contrast, higher concentrations promote the formation of selenopersulfide intermediates, which trigger oxidative stress and toxic effects [29].

Assuming selenium nanoparticles (SeNPs) exhibit minimal adverse effects, flavonoids may counteract these effects due to their ability to interact with SeNPs through hydroxyl (OH) groups on their rings, thereby enhancing their protective properties. The combination of flavonoids and nanoparticles improves interface interactions and surface characteristics in drug delivery systems [30]. However, flavonoids such as Myricetin and Baicalin suffer from poor water solubility, which poses a significant challenge to efficient drug absorption. One potential strategy to address this issue involves loading flavonoids into a water-soluble carrier material. When flavonoid solubility is enhanced via incorporation into SeNPs, the resulting nanoparticles gain improved water solubility. Additionally, the presence of flavonoids alters the conformational structure of bovine serum albumin (BSA), increasing its dispersion and thereby expanding surface area and interaction sites [31]. In the case of Baicalin and Myricetin, hydrophobic interactions play a critical role in their binding mechanisms. The interaction of these compounds with bovine serum albumin (BSA) induces subtle alterations in BSA’s secondary structure [32]. In this study, Baicalinaddressetin (BM) were co-encapsulated into selenium nanoparticles to address the limited bioavailability of BM and enhance its neuroprotective potential. This research offers new insights into the application of selenium-based nanoparticles carrying phytochemicals (baicalin and myricetin) in mitigating Aβ1-42-induced toxicity using an Alzheimer’s disease (AD) model.

## 2. Results and Discussion

### 2.1. Characterization of BM/Se@BSA NPs

#### 2.1.1. UV/Vis Spectrophotometer

**Figure 1:**
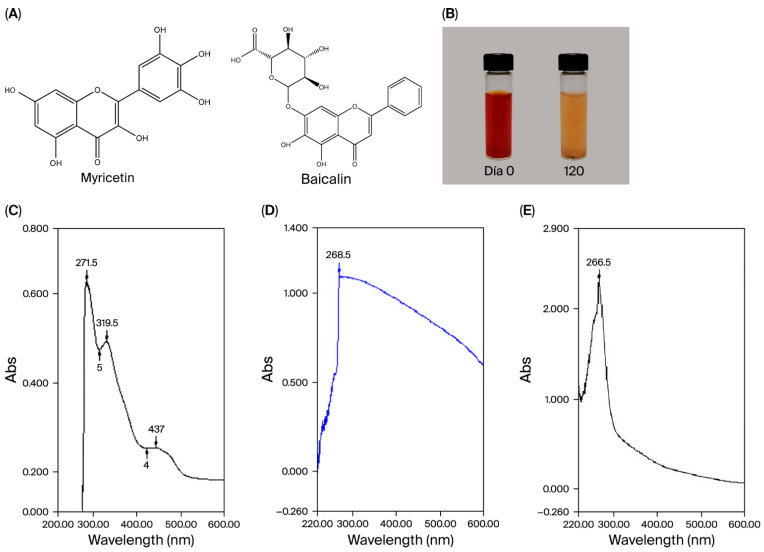
Chemical structure of Baicalin and Myricetin (**A**); stability over 170 days (**B**); UV–visible spectra of BSA (**C**), Se@BSA (**D**), BM (**E**), and BM/Se@BSA, n = 3.

The chemical structures of compounds baicalin and myricetin are presented. At the onset of the reaction, the solution exhibited a pale-yellow hue, which gradually deepened to an intense red (Figure 1B). This color transformation is attributed to the excitation of surface plasmons in selenium (Se) nanoparticles, indicating the successful reduction of H_2_SeO_3_ to elemental Se. Figure 1B also demonstrates that these Se nanoparticles-maintained stability in aqueous solution for up to 170 days, displaying a transparent appearance characteristic of well-dispersed nanoparticles. Stability was additionally assessed via Zeta potential measurements.

UV-visible spectrophotometric analysis revealed distinct absorption bands for compound BM at 271, 319, and 437 nm (Figure 1C). In Figure 1D, an absorption peak at 267.5 nm corresponds to the surface plasmon resonance of Se within Se@BSA nanoparticles. Additionally, a peak observed at 266.5 nm confirms the formation of BM/Se@BSA nanocomplexes (Figure 1E). The UV absorption centered at 266.5 nm further substantiates the successful adsorption of BM onto the nanoparticle surface.

#### 2.1.2. Intermolecular Interaction

**Figure 2:**
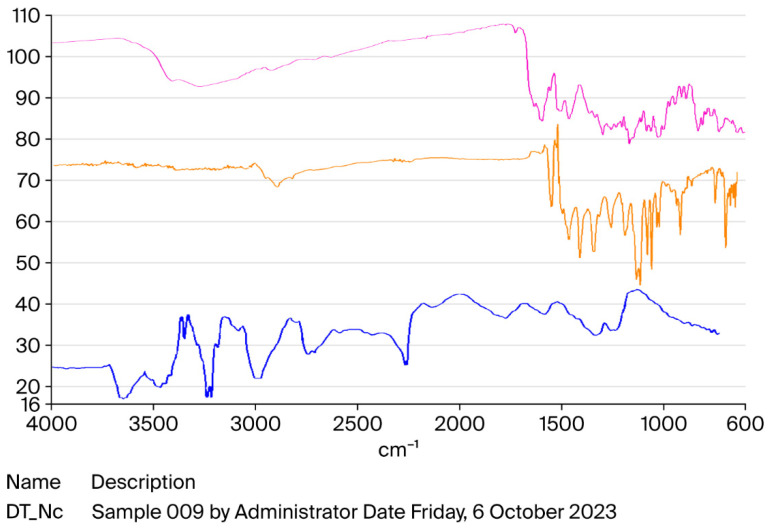
FT-IR spectra of (Pink) Se@BSA, (Orange) BM, and (Blue) BM/Se@BSA nanoparticles (NPs), n = 3.

Results obtained using FTIR spectroscopy were employed to investigate the intermolecular interactions between flavonoids and nanoparticles (Figure 2 pink). Measurements were recorded across the spectral range of 400–4000 cm^−1^. The BM sample exhibited several characteristic absorption peaks: hydroxyl group at 3267 cm^−1^, carbonyl group at 1756 cm^−1^, and additional peaks at 2925, 1656, 1594, 1517, 1462, 1376, 1335, 1298, 1199, 1181, 1167, 1084, 1027, 830, and 722 cm^−1^. Key spectral assignments include: 3267 cm^−1^: OH stretching vibrations, 2925 cm^−1^: CH_2_ and aliphatic C–H stretching, 1756 cm^−1^ and 1656 cm^−1^: C=O stretching, typical of flavonoid structures, 1594 cm^−1^ and 1517 cm^−1^: Aromatic C=C stretching vibrations, indicative of benzene rings, 1335 cm^−1^: CH_2_-related stretching vibrations. 1199 cm^−1^ and 1027 cm^−1^: C–O stretching. Among these, the absorptions in 1756, 1656, 1462, and 1335 cm^−1^ are particularly characteristic of flavonoids (Figure 2 pink), confirming their interaction with nanoparticles.

In this study, FT-IR spectroscopy was employed to investigate the interactions between BM and Se@BSA in the formation of BM/Se@BSA nanospheres. The Se@BSA spectrum displays a pronounced hydrogen bonding interaction (Se–OH), evidenced by the band at 3310 cm^−1^, corresponding to H–OH deformation vibrations of water. Characteristic peaks at 1750, 1647, 1635, 1350, and 1156 cm^−1^ suggest the successful synthesis of selenium nanoparticles stabilized with BSA (Figure 2 orange).

In the BM/Se@BSA composite, the emergence of absorption bands at 1623, 1553, 1445, 1442, 1303, 1249, 1199, and 1143 cm^−1^ indicates that multiple –OH groups from BM are conjugated with selenium, disrupting native BM hydrogen bonding and forming new C–O·Se interactions (Figure 2 blue). The FT-IR spectrum of BM differs significantly from that of Se@BSA, confirming that BM is not simply mixed with Se@BSA, but instead forms a stable conjugate by binding directly to the surface of selenium nanoparticles. FT-IR fingerprint analysis offers highly specific insights into the functional groups present in bioactive compounds. Each molecule possesses distinctive absorption bands in the infrared region, and the observed spectral differences between Se@BSA, BM, and BM/Se@BSA clearly demonstrate the formation of the BM/Se@BSA nanostructure.

#### 2.1.3. Particle Size Distribution Using Zeta Potential and Dynamic Light Scattering

**Figure 3:**
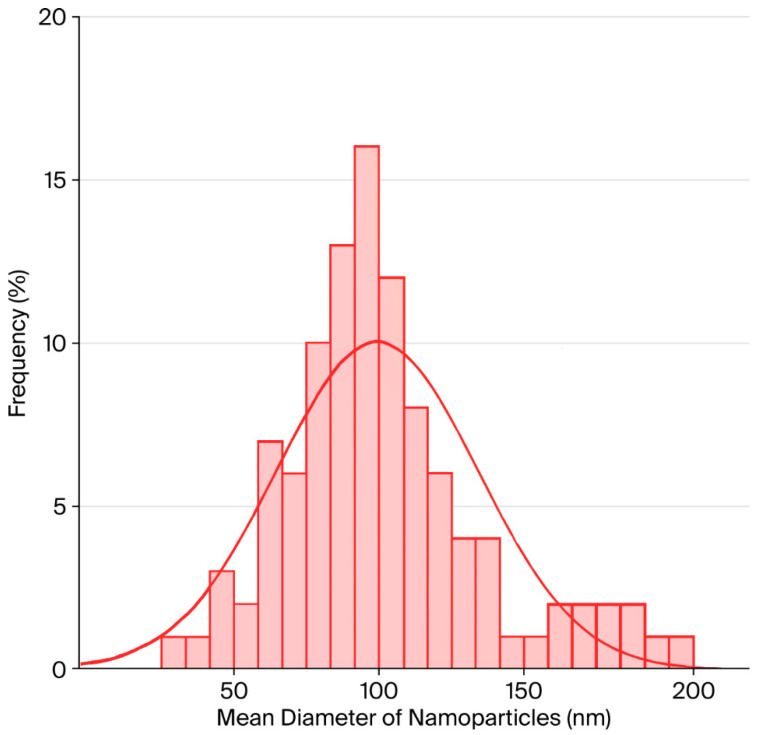
Size distribution of BM/Se@BSA.

Red elemental selenium was synthesized by reducing bulk selenium to its nanoscale form. Dynamic light scattering (DLS) was employed to determine the average particle size, polydispersity index (PDI), and zeta potential of selenium nanoparticles Se@BSA and BM/Se@BSA (Figure 3). The results revealed that BM/Se@BSA nanoparticles exhibited a negative surface charge, which contributed to their colloidal stability. The DLS measurements of the selenium nanoparticle dispersion showed an average particle size of 90.57 nm, a zeta potential of −27.2 mV, and a PDI of 0.270 for the BM-loaded formulation, indicating a uniform size distribution in the aqueous medium. These findings agreed with data obtained from transmission electron microscopy (TEM).

BM was successfully conjugated onto selenium nanoparticles (SeNPs) and characterized through various spectroscopic techniques. The resulting BM/Se@BSA nanocomposite displayed nanoscale dimensions, high stability, a predominantly amorphous nature, and a spherical morphology. To evaluate its hydrodynamic size and stability, dynamic light scattering (DLS) and zeta potential analysis were conducted. The zeta potential, which reflects the effective surface charge of nanoparticles, provided insights into their colloidal stability. An increase in zeta potential magnitude was associated with enhanced particle stability, attributed to stronger electrostatic repulsion between nanoparticles.

#### 2.1.4. Morphological Characterization of BM/Se@BSA Using Transmission Electron Microscopy

**Figure 4:**
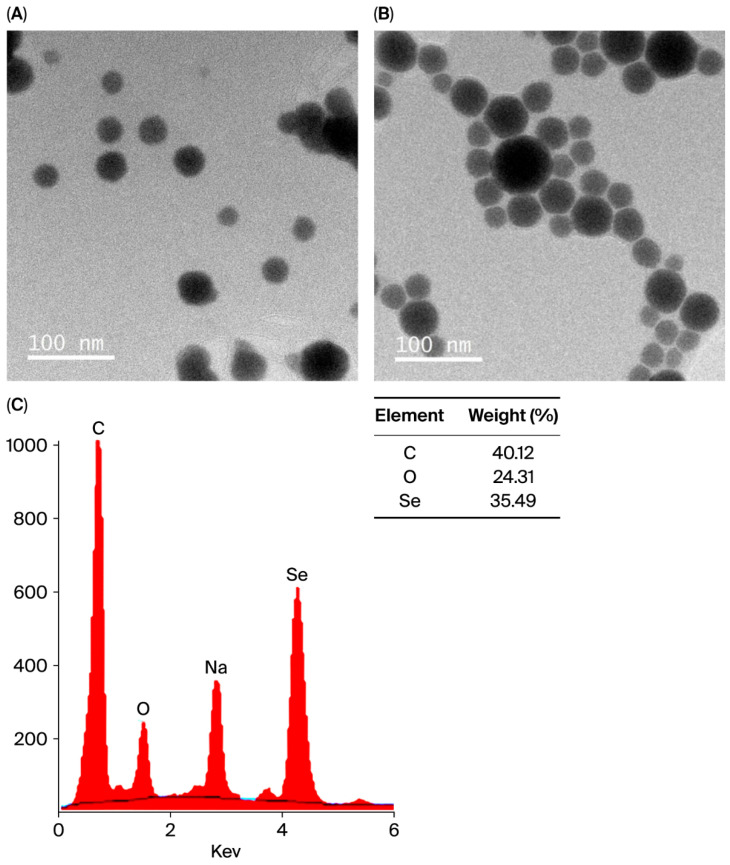
Images of synthesized nanoparticles: (**A**) Se@BSA (50 nm); (**B**) BM/Se@BSA (50 nm). Images were taken at 15,000× magnification with a scale bar of 50 nm. (**C**) X-ray diffraction analysis of BM/Se@BSA.

The high-resolution TEM image of Se@BSA nanoparticles displayed a well-defined spherical morphology (Figure 4A). Similarly, BM/Se@BSA particles also exhibited a predominantly spherical shape with no visible agglomeration (Figure 4B). Notably, the TEM analysis revealed marked differences in the chemical composition between untreated Se nanoparticles and those loaded with flavonoids.

#### 2.1.5. X-Ray Diffraction (XRD) Analysis of BM/Se@BSA

The XRD spectrum (Figure 4C) of the biosynthesized BSa/SeNPs displays a prominent peak at 1.5 keV, indicating the complete presence of selenium within the synthesized selenium nanoparticles. Figure 4C illustrates the X-ray diffraction analysis of the BM/Se@BSA crystal structure, revealing characteristic peaks corresponding to its elemental composition. Elements such as carbon (C), oxygen (O), sodium (Na), and selenium (Se) were identified. Notably, selenium accounts for approximately 24.25% of the mass, confirming the successful transformation from its bulk to nanoscale form. The clarity of the diffraction signals affirms the pure crystalline nature of BM/Se@BSA. Among the detected elements, carbon was found in the highest proportion (40.12%), followed by selenium (35.49%) and oxygen (24.31%) by weight. Thus, X-ray diffraction (XRD) analysis verified the presence of a pure crystalline phase in BM/Se@BSA.

#### 2.1.6. Encapsulation Efficiency (EE, %) and Loading Capacity (LC, %) of BM in BM/Se@BSA

**Figure 5:**
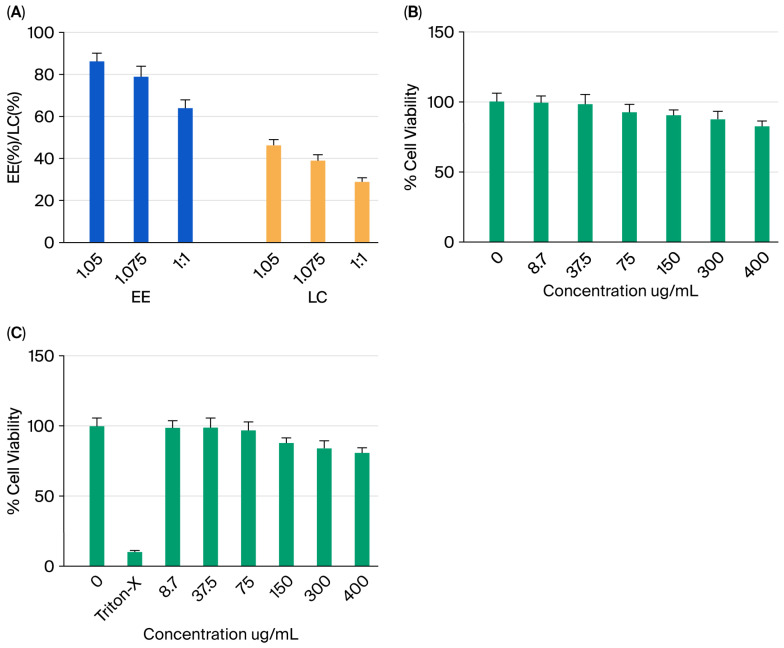
(**A**) Encapsulation efficiency and loading capacity of selenium/BM at ratios of 1:0.5, 1:0.75, and 1:1. (**B**) Cell viability was evaluated in RAW 264.7 cells then 24 h treatment to varying doses of BM/Se@BSA. (**C**) Evaluation of BM/Se@BSA-induced cytotoxic effects at different dosage levels. Results are presented as relative values (%) compared to the control ± SD.

The percentage of BM encapsulated within selenium nanoparticles (NPs), known as encapsulation efficiency (EE), is associated with the ratio between BM and Se@BSA, which depends on the solubility of BM in the matrix material. In our study, the EE reached 88.7% for a ratio of 1:0.5, 79.8% for 1:0.75, and 69.2% for 1:1 (Figure 5A). The highest efficiency was observed at the selenium/BM ratio of 1:0.5. Notably, EE increases as the weight ratio of selenium to BM decreases. This improvement is due to BM saturation in the system, which prevents further adsorption by Se@BSA, causing excess BM to be rapidly released during centrifugation.

Loading capacity (LC), which refers to the amount of BM encapsulated per unit weight of SeNPs, is significantly influenced by the molecular weight of the encapsulated compounds. This leads to a reduced LC at higher BM concentrations, with values of 51.2% (1:0.5), 39.4% (1:0.75), and 29.1% (1:1) (Figure 5A). Overall, the results highlight the excellent delivery capacity of the BM/Se@BSA system.

### 2.2. Cell Viability Assay of Nanoparticles in RAW 264.7 Cells and PC12 Cell Line

As shown in Figure 5B, MTS assays revealed an 18% reduction in viability between non-pretreated and pretreated cells. Notably, cell confluence remained stable even after 24 h of incubation, suggesting that BM/Se@BSA nanoparticles at concentrations of 300 and 400 µg/mL do not significantly impair cell growth.

The viability of PC12 cells exposed to BM/Se@BSA nanoparticles was further assessed across a concentration range of 8.7–400 µg/mL (Figure 5C). Cells treated with concentrations between 0.7 and 75 µg/mL-maintained viability comparable to untreated controls. However, notable decreases in viability were observed at the highest concentrations (300 and 400 µg/mL), potentially due to elevated selenium content and lower initial cell seeding density. Collectively, these results highlight the favorable biocompatibility of BM/Se@BSA nanoparticles, marked by low cytotoxicity and efficient cellular uptake.

### 2.3. Effect of BM, Se@BSA and BM/Se@BSA on Behavioral Condition in Treated Aβ1-42 Mice

To assess whether BM, Se@BSA, and BM/Se@BSA treatments can restore memory and cognitive function, we administered these compounds to Aβ1-42-induced Alzheimer’s disease (AD) model mice for 8 weeks. Following treatment, we conducted a series of behavioral tests—including the T-maze test to evaluate spatial learning and memory deficits, the open-field test to assess exploratory and locomotor activity, and the object recognition test to measure contextual memory. Aβ1-42 induction in AD mice is known to trigger spatial and contextual memory impairments, which can be reliably quantified through these testing paradigms.

Flavonoids are widely recognized for their neuroprotective potential, without causing adverse central nervous system (CNS) effects. Their benefits have been demonstrated in various behavioral assessments including the T-maze, Open Field Test, and Object Recognition Task [33]. These compounds are believed to enhance cognitive function by protecting neurons, stimulating neuronal activity, and promoting neurogenesis [34]. Following oral administration, flavonoids can cross the blood–brain barrier and have been detected in rat brain regions associated with learning and memory. Within the brain, their mechanisms of action include neural protection, facilitation of neuronal regeneration, and the stimulation of neurogenesis. Such neurobiological effects may help prevent memory decline related to aging and reduce the risk of neurodegenerative conditions, including different forms of dementia [35]. Human studies on flavonoids consistently report cognitive benefits, particularly in executive function, working memory, general memory performance, and processing speed.

Baicalin, a flavonoid known for its anti-apoptotic, antioxidant, and anti-inflammatory properties, shows high brain bioavailability when administered intranasally. Studies have revealed that approximately 52.36–100% of intranasally delivered baicalin reaches the brain via the olfactory pathway [36]. Another flavonoid, myricetin, has demonstrated efficacy in improving behavioral performance and maintaining hippocampal neuronal integrity. It achieves this by modulating oxidative stress, reducing neuroinflammation, and alleviating endoplasmic reticulum stress [37].

#### 2.3.1. Effect on Space Perceptive Ability

**Figure 6:**
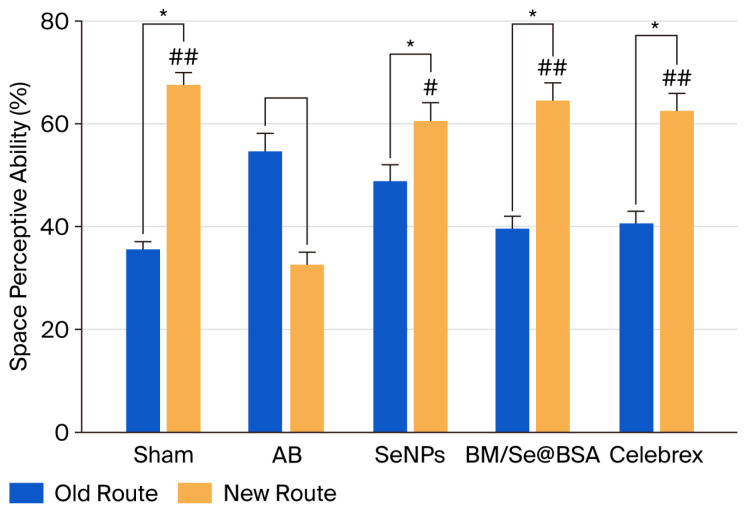
Effect of BM/Se@BSA on the T-maze test. Results are presented as mean ± SD (n = 8). Determination of abilities with old and new routes using Student’s *t*-test (* *p* < 0.05). Symbols indicated a statistically significant difference (^#^ *p* < 0.05, ^##^ *p* < 0.001).

T-maze was utilized to investigate cognitive abilities and memory of mice in space exploration and to assess the protective effect of BM, Se@BSA, and BM/Se@BSA on common behavioral dysfunction in Aβ1-42-induced memory impairment mice. The Aβ1-42 induced AD (control group), the exploration rate for new routes was 32%. However, the BM, Se@BSA, and BM/Se@BSA groups exhibited higher rates of exploration for the new route, specifically, 50 and 64%, respectively (Figure 6). Thus, the T-maze test was used to assess memory deficits in AD-like conditions in mice by examining brain function. BM/Se@BSA produced a higher spatial exploration rate for the new route than those observed in the Aβ1-42, control, and Celebrex treatment groups. Results demonstrated that nanoparticles have a protective effect on spatial memory deficits in C57BL6/J.

To assess the impact of BM/Se@BSA on two distinct forms of memory, cognitive locomotor memory and spatial learning—this study utilized the T-maze paradigm. Administration of Aβ1-42 significantly increased step-through latency during the memory retention trial, indicating impaired reference memory and spatial learning performance. Conversely, during the trial sessions, treatment with BM/Se@BSA s led to a notable reduction in escape latency, improved cognitive function, and mitigation of memory deficits associated with neuroinflammatory processes [38]. These findings suggest that Aβ1-42 administration impairs long-term spatial memory, as evidenced by prolonged escape latency. Importantly, BM/Se@BSA treatment effectively shortened escape latency without altering movement velocity, highlighting its potential to restore long-term memory deficits induced by Aβ1-42.

#### 2.3.2. Nanoparticles Enhanced Cognitive Abilities of AD Mice, in Open Field Test

**Figure 7:**
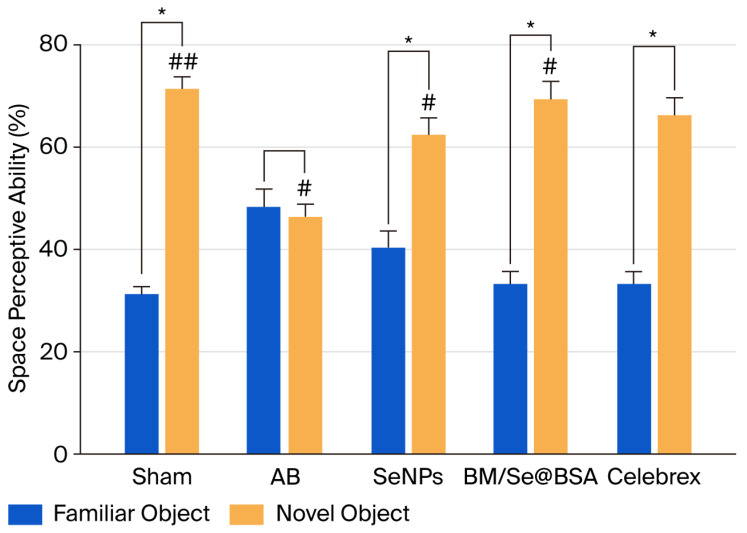
Effect of BM/Se@BSA on novel object recognition test. Determination of abilities in object recognition between familiar and novel objects. The spatial perceptive capacity assesses as the percentage of recognition interactions with each object above 5 min. Results are presented as mean ± SD (n = 8) and were analyzed using two-way ANOVA with Tukey’s test. * *p* < 0.05, ^#^ *p* < 0.05, ^##^ *p*< 0.001 If groups share the same symbols, it means their differences are not statistically significant.

**Figure 8:**
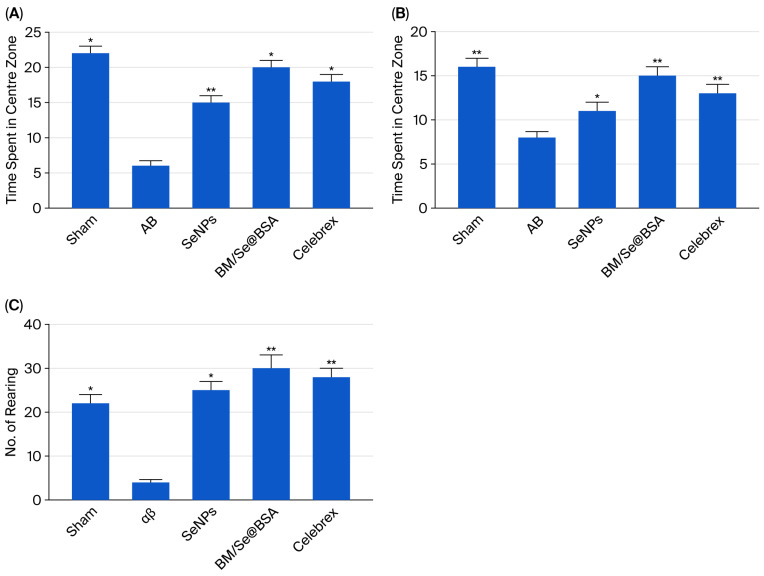
Effect of BM/Se@BSA in the open field test above 5 min: (**A**) minutes spent by mice in the center zone; (**B**) number of entries into the center in minutes; (**C**) number of line crossings, frequency of rearings. Results are presented as mean ± SD (n = 8) and were analyzed using two-way ANOVA with Tukey’s test. * *p* < 0.05, ** *p* < 0.01. If groups share the same asterisk, it means their differences are not statistically significant.

In this study, mice with Alzheimer’s disease (AD) induced by Aβ1-42 were used to investigate the effects of Se@BSA, and BM/Se@BSA in Open Field Test. The AD mice exhibited a significant increase in disorderly movements without any purpose in the open field test, particularly around the central field region (as shown in Figure 7). To evaluate behavioral responses to open spaces, animals were observed for five minutes in an open field arena. Mice in the control group displayed increased post-exposure activity, reflected by reduced occupancy of the central maze zones. Intergroup analysis revealed significant differences in time spent within the central zone: Celebrex-treated mice exhibited a marked increase (*p* < 0.01), while those receiving αβ treatment showed a notable reduction (*p* < 0.05 vs. control; Figure 8A). Central zone entry frequency also varied significantly among groups. Control mice made few entries into the center, whereas both BM/Se@BSA (*p* < 0.05) and Celebrex (*p* < 0.001) treatments led to substantial increases (Figure 8B). Locomotor activity following extended BM/Se@BSA exposure was assessed via line crossings and rearing events, both of which were significantly elevated compared to controls (*p* < 0.01; Figure 8C).

Additionally, the results of the Open Field Test in our study support the locomotor activity of BM/Se@BSA at dose 30 mg/kg. Mice treated with nanoparticles exhibited a significant improvement in their behavior, as evidenced by a higher number of crossings in the central area and less time spent along the outer edges of the field. Significant individual differences in locomotor activity were observed across groups, indicating that BM/Se@BSA administration affected sensorimotor performance. Therefore, the behavioral changes observed in the T-maze task were most likely attributable to enhanced memory function, rather than variations in sensorimotor abilities, motor output, or limb flexibility.

#### 2.3.3. Effect on Object Recognition Ability

During the training session, the rodent did not exhibit significant recognition of the two identical objects (A, A′). After 24 h, one of these was replaced by a novel object (B), resulting in the arrangement (A, B). In the normal control group, mice showed a natural tendency to explore the novel object more than the familiar one. In contrast, mice in the Alzheimer’s disease (AD) control group displayed similar levels of curiosity toward both objects (as shown in Figure 8), indicating an inability to discriminate between familiar and novel stimuli. This lack of distinction suggests cognitive impairment in the Aβ1-42-treated mice. Remarkably, treatment with BM, Se@BSA, and the combined formulation BM/Se@BSA led to improved recognition behavior. Specifically, mice treated with BM/Se@BSA demonstrated a stronger inclination to explore novel objects compared to those in other treatment groups. Mice in this group exhibited enhanced recognition and discrimination capabilities compared to the untreated Aβ1-42 group, highlighting the therapeutic potential of BM/Se@BSA in reversing episodic memory dysfunction.

The novel object recognition (NOR) test was employed to evaluate both short- and long-term memory. This study investigated the memory-enhancing potential of BM/Se@BSA against Aβ1-42-induced memory impairment in mice. In the short-term memory assessment, no significant differences were observed during the sample phase, where mice explored identical objects. However, in the test phase, mice treated with BM/Se@BSA at a dose of 30 mg/kg spent significantly more time exploring the novel object compared to the familiar one, indicating enhanced short-term memory. In the long-term memory evaluation, treated mice also showed a marked preference for the novel object during the test phase. These findings suggest that BM/Se@BSA nanoparticles may be effective in mitigating Aβ1-42-induced cognitive deficits, improving both short- and long-term memory performance in mice.

### 2.4. Alterations of the Morphologies of Amyloid Aβ1-42

**Figure 9:**
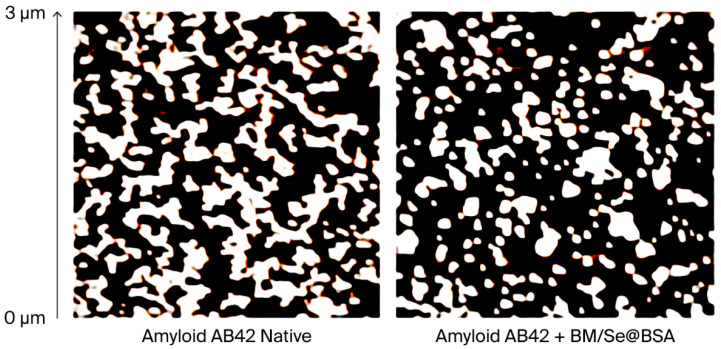
AFM images showing morphological variations of amyloid Aβ42 (native) after incubation with BM/Se@BSA at 37 °C for 24 h.

Atomic force microscopy (AFM) was employed to assess the morphological changes in amyloid structures incubated with BM/Se@BSA, as shown in Figure 9. Amyloid β peptide (Aβ42) alone served as the control and exhibited characteristic fibrillation after 72 h of incubation, the control group showed the formation of small oligomers. In contrast, treatment with BM/Se@BSA markedly reduced oligomer content. These results indicate that BM/Se@BSA effectively inhibits amyloid oligomerization by forming complexes that interfere with their structural development.

Flavonoids have demonstrated the ability to inhibit β-amyloid and Tau protein aggregation, neutralize free radicals, and chelate metal ions at clinically low concentrations [39]. Myricetin has been shown to enhance learning and memory in rat models of Alzheimer’s disease (AD) and exhibits pronounced antiamyloidogenic properties. It interferes with the formation of ordered Aβ aggregates by establishing hydrogen bonds between its hydroxyl groups and the carbonyl groups on β-sheet surfaces [40]. This interaction destabilizes interstrand hydrogen bonding, thereby hindering Aβ fibril elongation and preventing toxic conformational changes [41]. Additionally, myricetin modulates the activity of α- and β-secretase enzymes, further supporting its role in mitigating amyloid pathology. Baicalin also contributes to neuroprotection by directly reducing Aβ production and promoting the non-amyloidogenic pathway in both in vitro and in vivo models [42]. In this study, nanoparticles were engineered to counteract Aβ-induced mitochondrial dysfunction a hallmark of neurodegenerative disorders like AD. The high hydroxyl group content of the flavonoids embedded in these nanoparticles likely enables robust interaction with amyloid proteins, effectively impeding conformational shifts and aggregation.

### 2.5. Effect of BM/Se@BSA on Microglia Activation in the Cerebral Cortex

**Figure 10:**
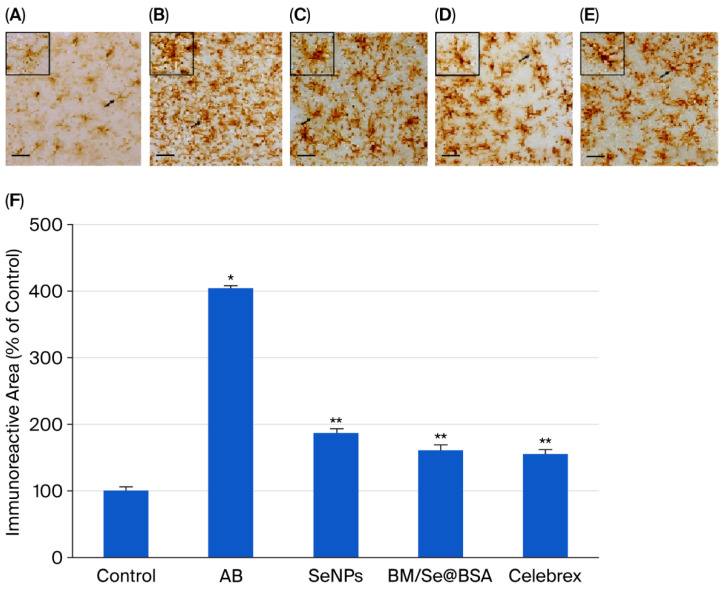
Effect of SeNPs and BM/Se@BSA on CD11b immunoreactivity in the mouse brain. (**A**–**E**) Photomicrographs of microglial activation distribution in the cerebral cortex via immunohistochemistry. (**F**) Bar graphs showing CD11b immunoreactive areas in mouse brains. Magnification: 400×, Scale bars: 100 μm. Values are presented as mean ± SD. Asterisks denote significance levels: * *p* < 0.001 and ** *p* < 0.01. Tukey’s post hoc test was used for pairwise comparisons. If groups share the same asterisk, it means their differences are not statistically significant.

CD11b expression is closely associated with microglial activation during neurodegenerative inflammation. Therefore, microglial activation was assessed through CD11b immunoreactivity. Photomicrographs (Figure 10A–E) illustrate the presence of CD11b-positive microglia in the brain. Exposure to Aβ1-42 peptide induced notable morphological changes such as shortened, thickened processes and elongated branches resembling an amoeboid shape indicative of activated microglia. This activation led to an increased presence of CD11b-positive cells compared to the control group (Figure 10D). In contrast, mice treated with Celebrex, SeNPs, and BM/Se@BSA (administered at 10 and 30 mg/kg, respectively) exhibited significant suppression of microglial activation compared to the Aβ1-42 group (Figure 10E). The quantification of CD11b immunoreactivity in the cerebral cortex region is shown in the bar graph (Figure 10F).

Microglial activation is a key feature of neuroinflammation, a pathological process commonly observed in both chronic and acute brain disorders. Numerous studies have highlighted the immunomodulatory potential of flavonoids across various diseases. Notably, flavonoids demonstrate the ability to attenuate microglial activity in the brains of aged mice, suggesting their potential to rejuvenate microglial populations and restore a more youthful neuroimmune profile. Flavonoids may regulate microglia both directly and indirectly, by dampening peripheral immune signaling to the brain [43]. For instance, baicalin has been shown to suppress microglial activation and inhibit inflammatory cytokine production in BV2 cells exposed to amyloid-beta (Aβ_42). It significantly reduced BV2 cell proliferation, lowered the expression of CD11b, and impaired the cells’ chemotactic capacity [44]. Similarly, myricetin appears to inhibit microglial activation, potentially slowing neurodegenerative processes. Its action is linked to a specific anti-STAT1 activity, resulting from direct interaction with the STAT1 protein [45].

### 2.6. Effects of BM/Se@BSA on IL-β, IL-6, TNF-α, and Il-10 Expressions

**Figure 11:**
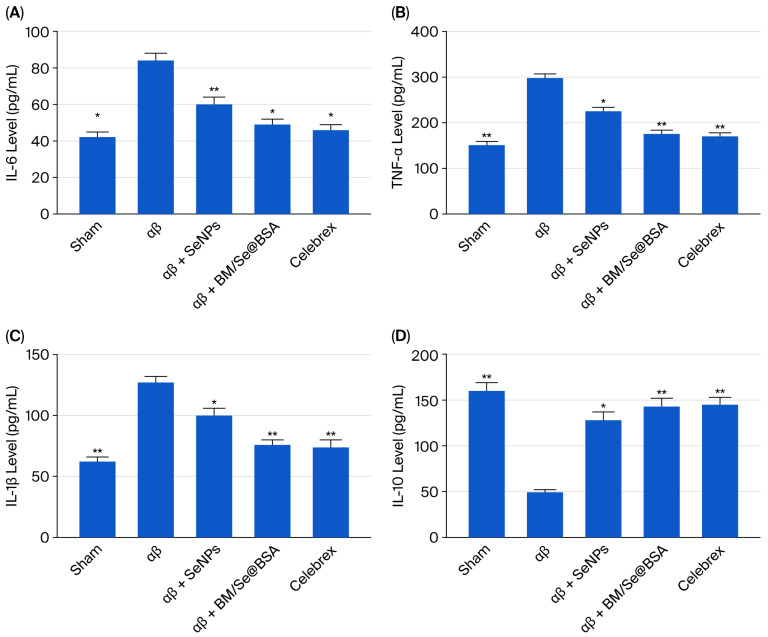
Effects of BM/Se@BSA in the brain of Aβ1-42-treated mice om IL-6 (**A**); TNF-α (**B**); IL-1B (**C**); IL-10 (**D**) expressions in the brain of the mice. Values show mean ± SD, the asterisk indicates the significant difference from Aβ1-42 group at * *p* < 0.05, ** *p* < 0.01.

The increase in pro-inflammatory cytokines, such as IL-1β and TNFα, is associated with cognitive deficits and neuroinflammation in AD. Effects of BM/Se@BSA on IL-1β and TNFα expression. Elevated levels of pro-inflammatory cytokines such as IL-β, IL-6, and TNF-α, are associated with cognitive impairment and neuroinflammation in Alzheimer’s disease (AD). The analysis was performed to assess the impact of BM/Se@BSA on IL-β, IL-6, TNF-α, and Il-10 expression in the cerebral cortex. Results indicate that Aβ1-42 peptide injection significantly increases both cytokines in the brain compared to the control group. Conversely, BM/Se@BSA and Celebrex significantly attenuated the expression of IL-6 (Figure 11A), TNF-α (Figure 11B), IL-β (Figure 11C). While increase Il-10 level (Figure 11D) in treated groups relative to the control and Aβ groups, highlighting their potential neuroprotective effects.

Moreover, sustained microglial activation initiates a proinflammatory cascade characterized by the release of key cytokines, including IL-1β and TNF-α. These molecules are well-established indicators of neuroinflammation, and elevated IL-1β levels have been consistently detected in the vicinity of Aβ plaques in Alzheimer’s disease (AD) brains. In this context, our results demonstrate that BM/Se@BSA markedly suppresses neuroinflammatory signaling, thereby alleviating cognitive impairments related to learning and memory in AD. Celebrex-treated rats also exhibited decreased levels of inflammatory markers in both brain regions. This aligns with previous studies reporting reductions in COX-2, IL-1α, IL-1β, IL-6, IL-12, and BDNF expression in rats treated with soluble amyloid-β (sAβ) and subsequently administered Celecoxib [46]. Consequently, Celecoxib is frequently employed as a positive control in experimental models of Alzheimer’s disease (AD) in rats.

Numerous studies have shown that phenolic phytocompounds not only combat neurodegeneration via free radical scavenging but also influence critical signaling pathways associated with neuroinflammation [47]. Flavonoids, in particular, demonstrate neuroprotective properties by reducing neuronal degeneration, primarily through the inhibition of lipoxygenases and cyclooxygenases [47]. Consequently, the neuroprotective effects of BM/SE@BSA are attributed to the anti-inflammatory potential of the phytocompounds baicalin and myricetin, whose therapeutic roles in neuroinflammation have been documented in previous research [48,49]. Nanoparticles exhibit significant inhibition of neuroinflammation; thus, it alleviates learning and memory dysfunction.

## 3. Materials and Methods

### 3.1. Selenium Nanoparticle Synthesis

Selenium nanoparticles (SeNPs) coated with serum albumin were synthesized via a one-step reaction sol–gel method involving selenous acid (H_2_SeO_3_), ascorbic acid, and bovine serum albumin (BSA). To prevent nanoparticle aggregation, BSA was incorporated as a stabilizing agent due to its excellent biocompatibility with selenium. Ascorbic acid functioned as the reducing agent. Specifically, 50 mM ascorbic acid was added dropwise to 1 mL of an aqueous solution containing H_2_SeO_3_ (100 mM) and BSA (10 mg/mL). Upon interaction between the reductant and selenous acid, the solution gradually shifted in color from light yellow to dark red, indicating nanoparticle formation. After 120 min of reaction, the SeNPs were collected by centrifugation at 18,000 rpm for 20 min and subsequently washed with distilled water and Milli-Q water (Merck Millipore, Burlington, MA, USA) [50].

### 3.2. Conjugation of BM with /Se@BSA

To synthesize BM/Se@BSA nanoballs, selenium nanoparticles (SeNPs, 100 mM) were mixed with a 25 mM solution of baicalin and myricetin in a 1:1 ethanol–water mixture. The resulting suspension was gently agitated for 1 h, then subjected to centrifugation at 16,000 rpm for 10 min. The collected precipitate was washed thoroughly with Milli-Q water, and the supernatant discarded. This washing step was repeated three times to remove residual impurities. Finally, the nanoball-containing solution was dialyzed against Milli-Q water for 24 h and stored at room temperature [50].

### 3.3. Characterization of SeNPs and BM/Se@BSA Nanoparticles

The size and polydispersity index (PDI) of the nanoparticles were determined via dynamic light scattering (DLS). Zeta potential measurements of both Se@BSA NPs and BM/Se@BSA NPs were carried out using a Zetasizer Nano ZSP (Malvern Instruments, Malvern, UK) at 25 °C, with all readings performed in triplicate. Surface morphology was examined using a high-resolution transmission electron microscope (TEM; Model HT7700, Hitachi High-Tech, Tokyo, Japan). The infrared spectra of the nanoparticles were recorded using a Bruker FTIR-TENSOR27 spectrophotometer (Ettlingen, Germany) across a wavelength range of 400–4000 cm^−1^. Additionally, UV-visible absorbance spectra were acquired with a Shimadzu UV-1800 spectrophotometer (Tokyo, Japan). Finally, elemental composition and chemical states were assessed via X-ray photoelectron spectroscopy (XPS) using a Thermo-Fisher ESCALAB 250Xi spectrometer (Waltham, MA, USA) equipped with a monochromatic Al Kα X-ray source.

#### 3.3.1. UV–Vis Spectrophotometer

The formation of selenium nanoparticles (SeNPs) was confirmed through UV–Vis spectrophotometric analysis (Waltham, MA, USA). To investigate the reduction of Se ions, a 1 mL aliquot of SeNPs was tested at three different concentrations. Measurements were recorded at 30 min intervals across a wavelength range of 200 to 600 nm, using Milli-Q water as the blank control [51].

#### 3.3.2. Fourier Transform Infrared (FTIR) Spectroscopy

The FTIR spectra indicated the presence of biomolecules potentially responsible for both the synthesis and stabilization of selenium nanoballs. These spectra also identified surface functional groups that may influence sorption mechanisms. For analysis, 2 mg of dried nanoparticle samples were examined, with scans conducted in the range of 1000 to 3500 cm^−1^ [51].

#### 3.3.3. Fourier Transform Infrared Spectroscopy

The Fourier transform infrared (FTIR) spectrum revealed potential biomolecules are sponsible for synthesizing and stabilizing Se nanoballs, as well as functional groups present on the nanoparticle surface that may play a role in sorption processes. For analysis, dried nanoparticle samples (2 mg) were used, and the FTIR scan covered a range between 1000 and 3500 cm^−1^ [51].

#### 3.3.4. Transmission Electron Microscope (TEM)

To assess the morphology of selenium nanoparticles (SeNPs), transmission electron microscopy (TEM) (Waltham, MA, USA) operating at 200 kV was employed. The nanoparticle solution was diluted with deionized water and sonicated for 10 min using a Vibronics VS 80 (Waltham, MA, USA). For electron micrograph acquisition, 2 mg of the sample were deposited onto carbon-coated copper grids and dried under vacuum for 30 min [51].

#### 3.3.5. Dynamic Light Scattering Spectroscopy

Additionally, the particle size distribution and the dispersion index of the nanoparticles revealed the distribution of droplet sizes, confirming their homogeneity. Sample, 3 mg of nanoparticles were analyzed using dynamic light scattering (DLS) at 25 °C. The laser attenuation filters were automatically adjusted during the measurements to optimize detection. This technique enabled determination of both the polydispersity index (PDI) and the average diameter of the nanoparticles. Particle size results were expressed as mean ± standard deviation [51].

#### 3.3.6. Energy Dispersive X-Ray Study

To verify the conversion of selenium ions to elemental selenium, an energy-dispersive X-ray (EDX) assay was conducted. A 1 mg sample was prepared following the same protocol used for transmission electron microscopy (TEM). The resulting TEM micrographs were subsequently analyzed using EDX (Waltham, MA, USA). The atomic percentages obtained through EDX provided insight into the elemental composition and purity of the synthesized nanoparticles [51].

#### 3.3.7. Encapsulation Efficiency (EE, %) and Loading Capacity (LC, %) of BM in Se@BSA Nanoparticles

BM-loaded Se@BSA nanoparticles were synthesized according to a previously reported method. A 40 mg sample of these nanoparticles was extracted in methanol (MeOH) using an ultrasound-assisted technique. The concentration of BM was determined by UV-visible spectrophotometry, using a standard curve measured at 319 nm on a UV-1800 spectrophotometer (Shimadzu Corporation, Kyoto, Japan). The encapsulation efficiency (EE, %) and drug loading capacity (LC, %) of BM were calculated using the following equations:

(1) $$Encapsulation\;efficiency\;EE(\%)=\frac{Amount\;of\;encapsulated\;BM\times 100\%}{The\;total\;amount\;of\;BM}$$

(2) $$Loading\;capacity\;(LC\%)=\frac{Amount\;total\;BM\;entrapped\times 100\%}{The\;total\;Selenium\;NPs\;weight}$$

### 3.4. Cytotoxicity of BM/Se@BSA

Ensuring the safety of nanoparticles is essential for the development of an effective drug. To evaluate this, the potential toxic effects of BM/Se@BSA were assessed using two methods. To assess the cytotoxic effects of BM/Se@BSA nanoparticles, RAW 264.7 macrophage cells (Sigma, St. Louis, MO, USA) were subjected to the MTT assay [3-(4,5-dimethylthiazol-2-yl)-2,5-diphenyltetrazolium bromide]. Cells were seeded at a density of 5 × 10^4^ per well in 96-well plates containing Dulbecco’s Modified Eagle Medium (DMEM; Gibco, Merck Kc, Darmstadt, Alemania), supplemented with 100 U/mL penicillin, 100 U/mL streptomycin, and 10% fetal bovine serum. Plates were incubated at 37 °C in a humidified atmosphere containing 5% CO_2_ for 24 h to achieve approximately 80% confluence. Following incubation, cells were treated with various concentrations of BM/Se@BSA nanoparticles (0, 18.7, 37.5, 75, 150, 300, and 400 µg/mL), based on previously reported dosages of selenium nanoparticles. Untreated cells served as the negative control, while cells exposed to 5% DMSO were used as the solvent control. After a 24 h exposure period, 20 µL of MTT solution (5 mg/mL) was added to each well and incubated for an additional 4 h. The medium was then removed, and 100 µL of DMSO was added to each well to dissolve the formazan crystals. Absorbance was recorded at 570 nm using a microplate reader.

In addition, cytotoxicity was evaluated using a luminescence-based assay. PC12 cell line, (1.5 × 10^4^ per well) were seeded into 96-well plates containing complete keratinocyte serum-free medium (SFM) and incubated for 24 h at 37 °C. After removing the medium, cells were treated with the same concentration range of BM/Se@BSA nanoparticles (0, 18.7, 37.5, 75, 150, 300, and 400 µg/mL). A 1% Triton X-100 (Dow Inc., Midland, MI, USA) solution was included as positive control. Plates were incubated for another 24 h at 37 °C. Subsequently, 200 µL of fresh medium and CellTiter-Glo (Promega, Madison, WI, USA) reagent were added to each well at a 1:1 (*v*/*v*) ratio. The plate was shaken on an orbital shaker at 300 rpm for 7 min, followed by a 10 min incubation at room temperature. Luminescence was then measured using a SpectraMax ID5 plate reader (Molecular Devices LLC, San Jose, CA, USA).

### 3.5. Experimental Animals and Protocol

Male C57BL/6J mice (obtained from bioterium of the National Polytechnic Institute, Mexico), aged 16 weeks and weighing between 30 and 38 g, were housed under standardized conditions: temperature maintained at 21–23 °C, relative humidity at 40–60%, and a 12:12 h light/dark cycle. Animals had ad libitum access to food and water throughout the study.

All experimental procedures were conducted in accordance with the “Principles of Laboratory Animal Care” (NIH Publication No. 85-23, revised 1985) and the Mexican Official Norm (NOM-062-Z00-1999). Ethical approval was obtained from the Institutional Ethics Committee of ENCB (Approval No. ENCB/CEI/059/2024; 10 January 2024).

Following a one-week acclimation period, mice (n = 50) were randomly assigned to five experimental groups (10 animals per group). Amyloid-β1-42 peptide (Enzo Life Sciences, Farmingdale, NY, USA; 1 μg/μL) was solubilized in 5% acetic acid and incubated at 37 °C for 24 h to promote aggregation. Mice were anesthetized via intraperitoneal injection of thiopental sodium (80 mg/kg body weight) and received bilateral injections of aggregated Aβ1-42 (1 μL per ventricle) at a rate of 0.2 μL/min into the lateral ventricles. The six treatment groups included the following:

Group I: normal
Group II: Aβ1-42 peptide
Group III: Aβ1-42 peptide + SeNPs (30 mg/kg)
Group IV: Aβ1-42 peptide + BM/Se@BSA (30 mg/kg)
Group V: Aβ1-42 peptide + Celebrex (10 mg)

BM/Se@BSA treatments were administered orally once daily for 8 weeks. Power analysis to determine the required nanoparticle dosage was conducted using GraphPad Prism software 10.6.0 Control mice received normal saline, and all behavioral assessments were performed between 10:00 and 15:00 by trained personnel. At the end of the experiments Mice were sacrificed, brain tissues (cortex) was excised and used for further studies.

### 3.6. Behavioral Studies

#### 3.6.1. T-Maze Test

Neurocognitive function was assessed using the rewarded T-maze test. Prior to the experiment, mice were trained by allowing them to freely explore the entire maze and receive a food reward upon task completion. The apparatus consisted of a T-shaped maze with a start box, a left arm, and a right arm. Each arm, as well as the starting and goal paths, measured 50 cm in length, 13 cm in width, and 20 cm in height. During the first trial, the left arm was blocked, and the mouse was placed in the start box to explore the maze for 10 min [30], after which it was returned to its cage. On the following day, both arms were accessible, and the animal was allowed to explore for another 10 min. The number of entries into each arm was recorded and subsequently analyzed [52].

#### 3.6.2. Novel Objection Recognition (NOR) Test

To assess the distinction between familiar and novel objects during the testing trial, we compared the exploration time spent on each. Discrimination was calculated as the proportion of time spent exploring the novel object relative to the total time spent exploring both objects during the trial. The experimental setup consisted of a rectangular box (50 × 50 × 25 cm). One day prior to testing, each mouse was allowed to freely explore the box for 5 min to become familiar with the environment. During the habituation phase, two identical objects (A and B) matched in color, texture, shape, and size were placed in the box. The mouse was allowed to explore the objects for 10 min, after which it was returned to its home cage. Two hours later, object A was replaced with a novel object C, positioned identically to the original placement. The mouse was reintroduced to the box and allowed to explore for 5 min. Object recognition was quantified by dividing the time spent exploring the novel object (C) by the total time spent exploring both objects (B and C) [52].

#### 3.6.3. Exploratory Behavior in Open-Field Test

The open-field test is a widely used behavioral assay to evaluate exploratory activity and general locomotion in rodents. In this study, mice were individually placed at the center of a cylindrical open-field arena (40 cm in diameter × 31 cm in height, white-colored) to assess spontaneous behavior over a 5 min session. Each mouse was allowed to explore the arena only once. Prior to testing the next animal, the maze was cleaned using 70% isopropyl alcohol to eliminate any residual scents that could influence behavior. Preference for the central area was evaluated by recording both the number of entries and the duration spent in the central zone. Additionally, the effects of nanoparticle administration on locomotor activity were assessed by quantifying the number of rearing and line crossings during a 5 min exploration period [53].

### 3.7. Atomic Force Microscopy (AFM) Assay

Atomic Force Microscopy (AFM) is a highly effective technique for visualizing and analyzing the nanoscale structural characteristics of amyloid fibrils. In this study, Aβ1-42 peptides were solubilized in 4 mM HEPES buffer and diluted to a final concentration of 120 μM. For Thioflavin T (ThT) fluorescence experiments, the Aβ1-42 solution (40 μM) was mixed with PP (10.0 μM) and incubated at 37 °C for 30 min. The resulting samples were deposited onto freshly cleaved mica disks (Ø 1 cm), rinsed with 200 μL of Millipore water (Merck Millipore, Burlington, MA, USA) and dried under a stream of nitrogen. AFM imaging was performed using a Nanoscope IIIa microscope (Veeco Instruments, Santa Barbara, CA, USA), with scan rates of 1–3 Hz over areas of 1–2 μm^2^ and a resolution of 514 × 514 pixels.

### 3.8. Brain Processing

Following the completion of behavioral assessments, the animals were anesthetized and perfused with 0.9% normal saline. The brains were promptly extracted, placed on ice, and sectioned before being immersed in a 30% sucrose solution. Subsequently, the tissues were fixed in an ice-cold 4% paraformaldehyde solution. Coronal brain sections (35 μm thick) were then obtained using a microtome and stored in a 0.01 M phosphate buffer solution at cold temperatures.

### 3.9. Immunohistochemistry

Immunohistochemistry is a laboratory technique used to detect specific proteins such as amyloids in tissue samples using targeted antibodies. For this procedure, coronal brain sections were collected from each mouse and incubated in phosphate-buffered saline (PBS) containing 1% bovine serum albumin (BSA) for 1 h. These sections were then incubated overnight with a mouse monoclonal anti-integrin M (CD11b) antibody (1:100; Merck, CA, USA). After rinsing, the sections were incubated with an HRP-conjugated goat anti-mouse IgG secondary antibody (1:500; Thermo Fisher, Waltham, MA, USA) at 25 °C for 2 h. Following another PBS rinse, the tissue sections were exposed to a PBS solution containing 0.001% diaminobenzidine tetrahydrochloride dihydrate (Sigma-Aldrich, St. Louis, MO, USA) and 0.003% hydrogen peroxide. The stained sections were mounted onto gelatin-coated glass slides, dehydrated overnight, and sealed with DPX mounting medium.

Microglia-positive cells within the coronal brain slides were counted using a light microscope at 40× magnification. Immunostaining results were analyzed with Java-based software 1.54p (Windows version; National Institutes of Health, Bethesda, MD, USA), and the data were expressed as the percentage of immunoreactive area relative to the control, calculated using the following formula:

$$Immunoreactive\;area=\frac{areas\;of\;CD11b-positive\;microglia\;(positive\;pixels)}{total\;area\;of\;theimage\;(total\;pixels)}$$

### 3.10. Enzyme-Linked Immunosorbent Assay (ELISA)

The levels of cytokines IL-β, IL-6, TNF-α, and Il-10 expressions in mouse brain homogenates were measured using ELISA, adhering to the same protocol. All samples were analyzed in duplicate and normalized to total protein content, determined via the BCA assay.

### 3.11. Statistical Analysis

Experimental results are presented as mean ± standard deviation (SD). Statistical analyses were performed using GraphPad Prism version 6.0 (GraphPad Software, Inc., La Jolla, CA, USA). Comparisons between two groups were evaluated using Student’s *t*-test for continuous variables. For comparisons involving multiple groups, one-way analysis of variance (ANOVA) followed by Tukey’s post hoc test was applied. Differences with *p* values less than 0.05 were considered statistically significant.

## 4. Conclusions, Limitations, and Future Directions

For several decades, a wide range of therapeutic strategies have been explored; however, current treatments primarily address symptoms rather than underlying causes. Based on this, we focused on the synthesis of BM@Se, which may offer new avenues to alleviate cognitive decline associated with Alzheimer’s disease (AD). In this study, Baicalin and Myricetin (BM) were integrated into selenium nanoparticles (Se-BM) to overcome BM’s low solubility and enhance its neuroprotective potential. Our findings show that Se-BM markedly mitigates amyloid aggregation, neural damage, and cognitive dysfunction in an Aβ1-42 -induced mouse model of neurodegeneration. Looking ahead, this study highlights the potential of selenium nanoparticles combined with BM as a promising approach for treating Alzheimer’s disease. Their ability to cross the blood–brain barrier positions them as strong candidates for clinical research, especially due to their role in minimizing toxicity anti-inflammatory properties and inhibiting amyloid beta peptide fibrillation.

Recent investigations highlight the promising role of nanoparticles in the treatment of Alzheimer’s disease (AD), largely due to their capacity to traverse the blood–brain barrier (BBB). This unique ability renders them effective candidates for inhibiting amyloid beta peptide fibrillation and reducing its neurotoxic effects. To advance this therapeutic strategy, there is a critical need for systematic studies focused on biodegradable nanomaterials that can be engineered to carry multiple disease-modifying agents, thereby improving treatment outcomes. Nanoparticle-based therapies represent an emerging and underexplored domain, particularly in the context of in situ neuronal transplantation or regeneration. These approaches may offer the potential to restore central nervous system (CNS) integrity and alleviate cognitive decline in AD patients. Additionally, nanoparticles provide a versatile platform for drug delivery across the BBB, facilitating targeted intervention within the CNS. Despite these encouraging developments, several challenges remain among them include achieving efficient and specific binding of nanoparticle-loaded therapeutics to amyloid plaques. Current research efforts are actively addressing these complexities. Within this framework, nanodrug delivery systems stand out as a promising avenue for modulating AD pathogenesis and advancing more effective therapeutic strategies. The limitations of this study are presented in Table 1 [54,55,56,57].

## Figures and Tables

**Table 1:** Limitations in this investigation as well as the proposed methodology to develop in future studies.

Limits	Methodology	References
Tau Aggregation	Tau Aggregation Inhibition Assay ThS Fluorescence Assay	[54] [54]
Antioxidant properties	Estimation of Oxidative Stress Markers (MDA, GSH, and NRF2)	[55]
Histopathology in Brain tissues	Hematoxylins and Eosin assay	[56]
Measurement of brain activity of AChE	5,5-dithiobis-2-nitrobenzoic Acid and Turns Yellow Assay	[57]

## Data Availability

All data of relevance are duly incorporated in the article.
